# Tumor Lysis Syndrome Induced by Selpercatinib in Rearranged During Transfection (RET) Fusion-Positive Non-Small-Cell Lung Cancer

**DOI:** 10.7759/cureus.98134

**Published:** 2025-11-30

**Authors:** Shinobu Sagawa, Yoshitaka Tomoda, Jun Shikama, Yukikazu Awaya

**Affiliations:** 1 Respiratory Medicine, Itabashi Chuo Medical Center, Tokyo, JPN; 2 General Internal Medicine, Itabashi Chuo Medical Center, Tokyo, JPN

**Keywords:** non-small cell lung carcinoma (nsclc), ret fusion, ret kinase inhibitor, selpercatinib, tumor lysis syndrome

## Abstract

Tumor lysis syndrome (TLS) is a rare but potentially fatal oncologic emergency in patients with solid tumors. Though well documented in hematologic malignancies, TLS in non-small-cell lung cancer (NSCLC) has been infrequently reported. We report the case of a 78-year-old woman diagnosed with stage IVB NSCLC harboring a KIF5B-rearranged during transfection (RET) fusion. Selpercatinib was initiated at 160 mg twice daily. At baseline, the patient had normal renal function and no classical laboratory risk factors for TLS. By day seven of treatment, biochemical abnormalities fulfilled the Cairo-Bishop criteria for laboratory and clinical TLS: uric acid rose to 13.3 mg/dL, phosphate to 5.4 mg/dL, and creatinine to 2.15 mg/dL. Selpercatinib was discontinued, and treatment with intravenous hydration, febuxostat, and furosemide was initiated. No arrhythmias or seizures occurred. Biochemical abnormalities resolved within five days. Selpercatinib was successfully resumed without recurrence of TLS. A partial radiographic response was observed during the following four months. To our knowledge, this is the first reported case of TLS induced by selpercatinib in RET fusion-positive NSCLC. This case highlights that potent targeted therapy can trigger TLS even in the absence of conventional risk factors. Early recognition and aggressive management are essential to mitigate risk and allow continuation of effective treatment.

## Introduction

Tumor lysis syndrome (TLS) is an oncologic emergency caused by rapid tumor cell destruction, resulting in electrolyte abnormalities and acute kidney injury. While TLS is well recognized in hematologic malignancies, its occurrence in solid tumors is uncommon [1]. However, the advent of highly effective molecular targeted therapies has transformed cancer treatment, achieving remarkable response rates of 57% or higher in molecularly defined malignancies [2]. This unprecedented efficacy may paradoxically increase the risk of TLS through rapid tumor regression, even in solid tumors previously considered at low risk. Indeed, TLS has been increasingly reported with the use of molecular targeted therapies. Therefore, standard monitoring after initiation of targeted agents typically includes baseline and periodic assessment of renal function, electrolytes, and tumor markers, though specific protocols vary by agent and clinical context. We report an unusual case of TLS following selpercatinib initiation in rearranged during transfection (RET) fusion-positive non-small-cell lung cancer.

## Case presentation

**Figure 1:**
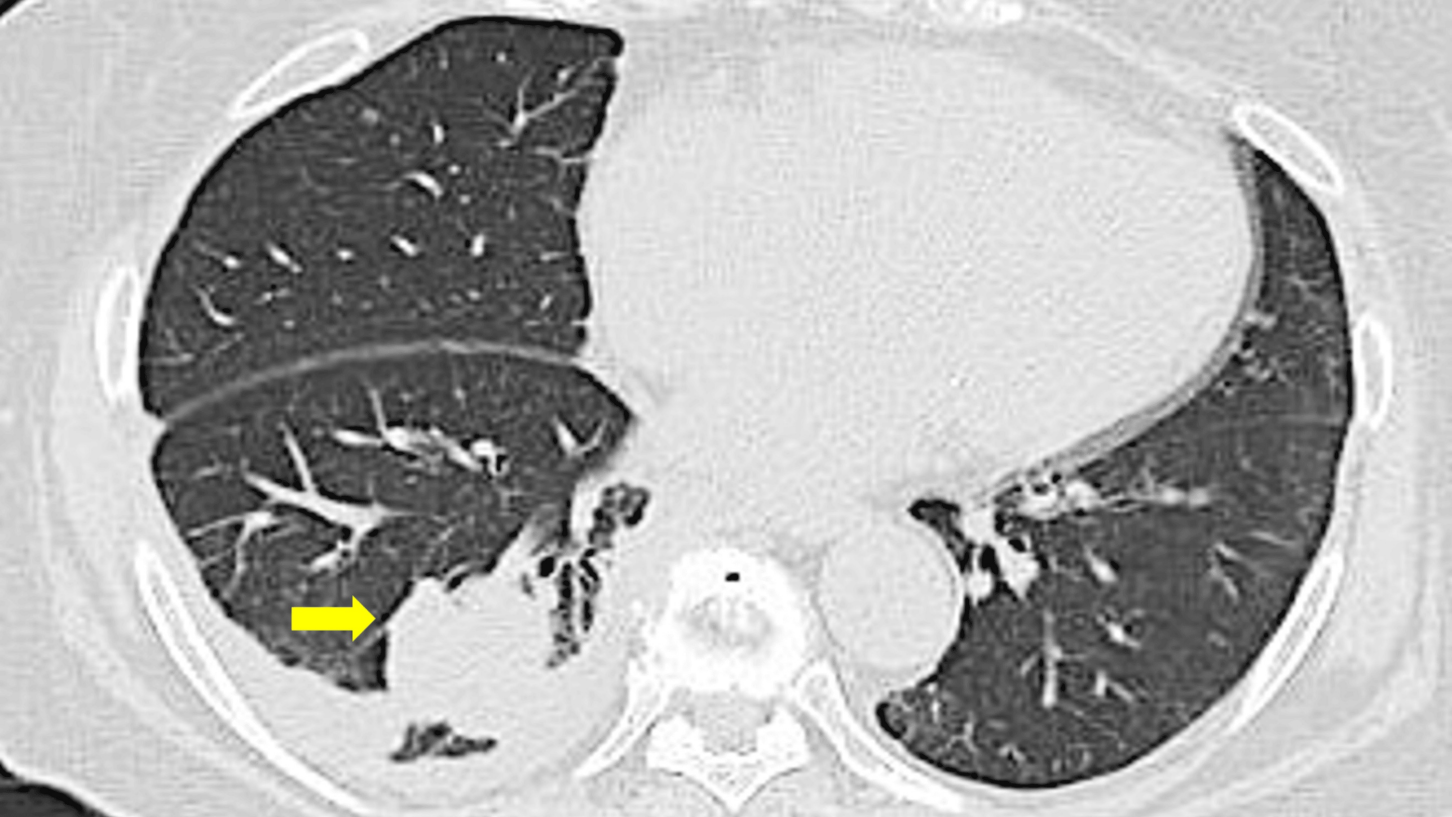
Chest computed tomography scan shows a 30-mm mass in the right lower lobe (arrow).

A 78-year-old Japanese woman with a medical history of hypertension, type 2 diabetes mellitus, and chronic heart failure was admitted for initiation of selpercatinib therapy. Two months earlier, she had undergone evaluation for an abnormal shadow on chest radiograph, which led to the discovery of a 30-mm mass in the right lower lobe on chest computed tomography (CT) (Figure 1).

**Figure 2:**
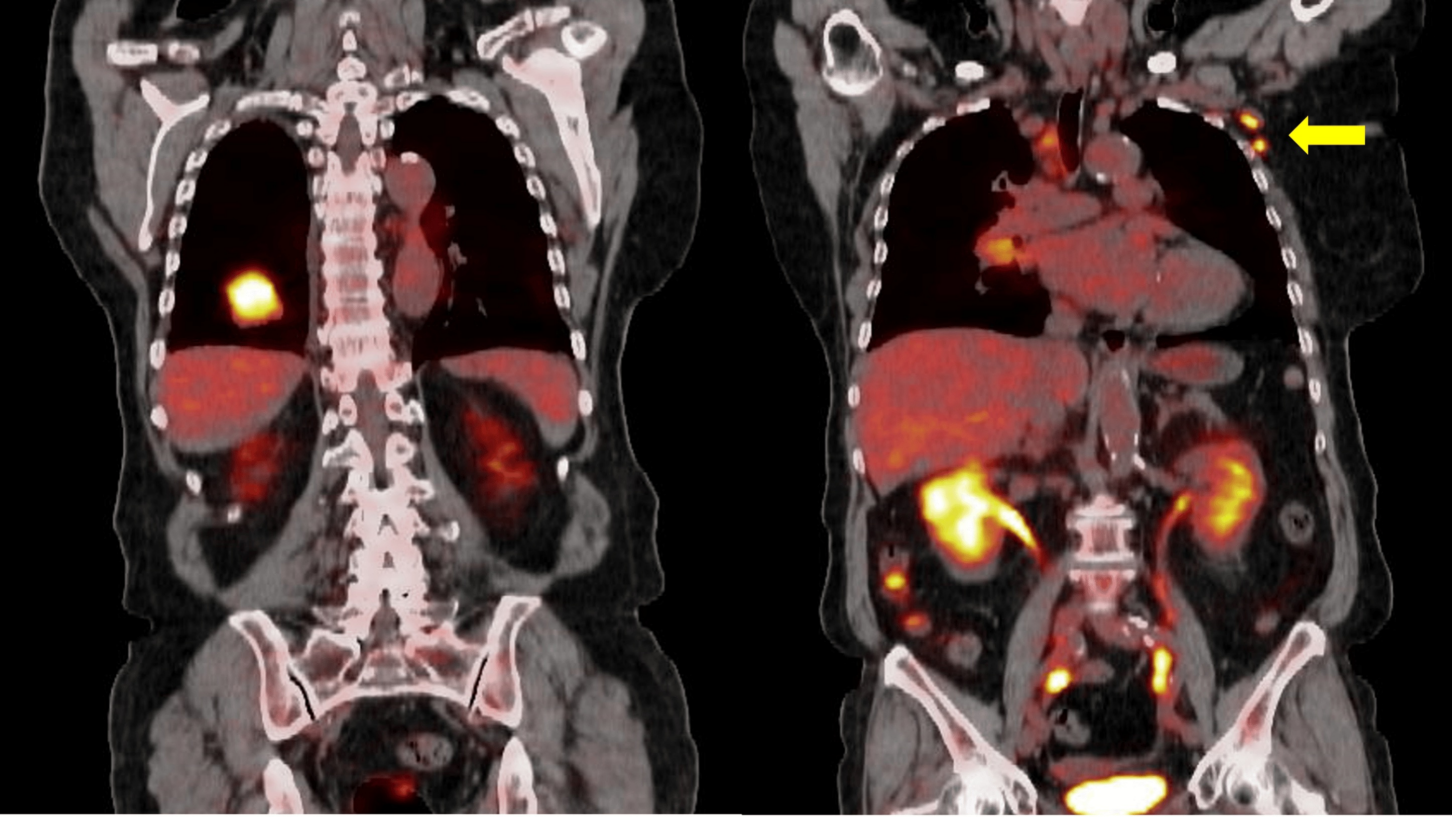
Coronal positron emission tomography/CT scan shows increased uptake of 18F-fluorodeoxyglucose to the tumor of the right lower lobe and left axillary lymph nodes (arrow).

A surgical lung biopsy revealed invasive adenocarcinoma. Staging with positron emission tomography/CT (PET/CT) showed increased uptake of 18F-fluorodeoxyglucose (FDG) to the right hilar, subcarinal, and left axillary lymph nodes, but no evidence of distant organ or bone metastases (Figure 2).

Molecular testing revealed a KIF5B-RET gene fusion, classifying her cancer as stage IVB RET-rearranged non-small cell lung cancer (NSCLC). At admission, laboratory values were as follows: creatinine 0.84 mg/dL, uric acid 5.6 mg/dL, phosphate 3.9 mg/dL, and lactate dehydrogenase (LDH) 342 U/L. The patient had preserved functional status (Eastern Cooperative Oncology Group (ECOG) performance status 1) and was asymptomatic. Selpercatinib was initiated at the standard dose of 160 mg orally twice daily. By day four of treatment, laboratory monitoring revealed increasing phosphate (5.0 mg/dL) and creatinine (1.35 mg/dL). On day seven, uric acid had risen to 13.3 mg/dL, phosphate to 5.4 mg/dL, and creatinine to 2.15 mg/dL. The patient remained asymptomatic but met the Cairo-Bishop criteria for both laboratory and clinical tumor lysis syndrome (TLS). Selpercatinib was promptly discontinued, and the patient was started on aggressive intravenous hydration (3000 mL/day of normal saline), oral febuxostat (20 mg/day), and intravenous furosemide (20 mg/day). Electrolytes were monitored every 24 hours. No arrhythmias or seizures occurred, and the patient did not require hemodialysis. By day 12, uric acid had decreased to 8.0 mg/dL, and creatinine had returned to 1.53 mg/dL (Table 1).

**Table 1:** Laboratory of data during the clinical course. BUN: blood urea nitrogen; Cr: creatinine; Na: sodium; K: potassium; Cl: chloride; UA: uric acid; P: phosphorus.

Parameters	Day of admission	Day 4	Day 7	Day 8	Day 12	Normal range
BUN (mg/dL)	16.9	21.7	37.7	34	20.9	8-20 mg/dl
Cr (mg/dL)	0.84	1.35	2.15	1.93	1.53	0.46-0.79 mg/dl
Na (mEq/L)	141	141	140	142	142	138-145 mEq/L
K (mEq/L)	3.7	3.9	3.5	3.8	3.1	3.6-4.8 mEq/L
Cl (mEq/L)	105	99	102	99	102	101-108 mEq/L
UA (mg/dL)			13.2	11.4	8	2.6-5.5 mg/dl
P (mg/dL)	3.9	5	5.4	4.3	3.5	2.7-4.6 mg/dl

**Figure 3:**
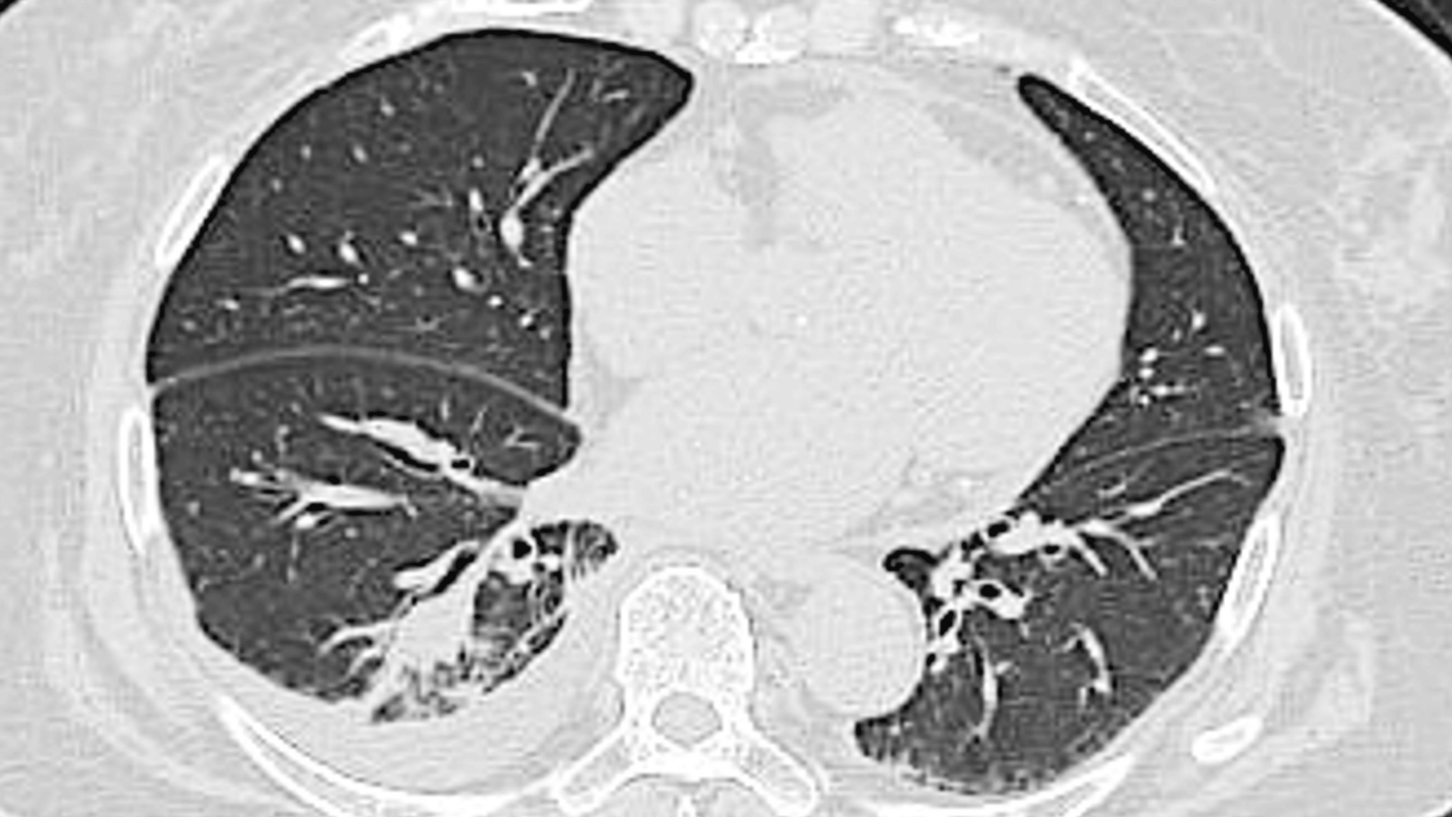
Chest computed tomography scan one month after selpercatinib administration shows a reduction in tumor size.

After multidisciplinary discussion, selpercatinib was cautiously reintroduced at full dose under close monitoring. No recurrence of TLS was observed, and the patient remained clinically stable with radiographic evidence of partial response to therapy over the following one month (Figure 3).

## Discussion

TLS is a life-threatening oncologic emergency that results from the rapid destruction of malignant cells, releasing intracellular contents such as potassium, phosphate, and nucleic acids into the bloodstream. The resulting electrolyte and metabolic disturbances can lead to acute renal failure, cardiac arrhythmias, seizures, and even death if not managed appropriately [3]. According to the widely accepted Cairo-Bishop criteria, TLS is diagnosed when two or more of the following laboratory abnormalities occur within three days before or seven days after the initiation of cytotoxic therapy: hyperuricemia (≥8.0 mg/dL or a 25% increase), hyperkalemia (≥6.0 mmol/L or a 25% increase), hyperphosphatemia (≥4.5 mg/dL or a 25% increase), and hypocalcemia (≤7.0 mg/dL or a 25% decrease). Clinical TLS includes these laboratory findings in conjunction with one or more clinical complications, such as renal dysfunction, seizures, cardiac arrhythmia, or sudden death [1]. In the present case, the patient developed significant biochemical changes: phosphorus increased from 3.9 to 5.4 mg/dL, uric acid rose to 13.3 mg/dL, and serum creatinine increased from 0.84 to 2.15 mg/dL within the first seven days of selpercatinib administration, thus fulfilling the Cairo-Bishop criteria for both laboratory and clinical TLS. These findings confirm the diagnosis and underscore the potential severity of TLS in patients with solid tumors receiving targeted therapy.

Although TLS is most commonly associated with hematologic malignancies, its occurrence in solid tumors has been increasingly reported [4]. The incidence remains low, but when TLS occurs in solid tumors, it carries a significantly higher mortality rate, reportedly reaching up to 54% [1]. The most commonly cited risk factors for TLS in solid tumors include high tumor burden, rapid cellular proliferation, elevated serum LDH, renal insufficiency, liver metastasis, and high sensitivity to cytotoxic or targeted therapy [1]. In the present case, TLS developed despite normal LDH levels and preserved renal function, suggesting that traditional risk stratification may be insufficient when initiating highly active molecular therapies.

RET gene fusions are identified in 1%-2% of NSCLC, more frequently in younger, never-smoking patients with adenocarcinoma histology [5]. Targeted therapies such as selpercatinib have dramatically altered treatment outcomes. Clinical trials demonstrated objective response rates exceeding 80% in treatment-naïve patients, with a median progression-free survival of over 17 months [6]. This high efficacy, while beneficial for disease control, may also increase the risk of rapid tumor breakdown and subsequent TLS. Notably, to date, only one prior case of TLS induced by selpercatinib has been reported, and that in the context of medullary thyroid carcinoma [7]. The rarity of selpercatinib-induced TLS is further supported by data from the pivotal LIBRETTO-001 trial. In the medullary thyroid carcinoma cohort of 531 patients, only one case of TLS was reported, while no cases of TLS were documented among NSCLC patients treated with selpercatinib [8]. This highlights the exceptional nature of the present case and underscores the unpredictability of TLS occurrence even with agents demonstrating high response rates across multiple tumor types. Notably, the reported medullary thyroid carcinoma case had renal dysfunction as a risk factor for TLS [7]. In contrast, our patient developed TLS despite preserved baseline renal function, further emphasizing the unpredictable nature of this complication in NSCLC. The present report is the first to describe TLS following selpercatinib administration in NSCLC.

The role of molecular targeted agents in TLS pathogenesis is gaining attention. A systematic review of TLS in solid tumors found that 8% of reported cases involved targeted therapies rather than conventional chemotherapy [1]. Agents such as afatinib and brigatinib have all been implicated [9,10]. These therapies often induce rapid tumor regression, particularly in tumors with driver mutations or high oncogene addiction, which may precipitate TLS even in patients without classical risk factors.

The Cairo-Bishop criteria remain the primary standard for defining and stratifying TLS risk [3]. The pathophysiology of TLS is primarily driven by the massive release of intracellular potassium and phosphate, and the catabolism of nucleic acids to uric acid. The precipitation of uric acid and calcium phosphate within renal tubules can result in acute kidney injury [3]. Management strategies include aggressive intravenous hydration, uric acid-lowering agents such as febuxostat or allopurinol, loop diuretics to maintain high urine output, and the use of rasburicase in cases of severe hyperuricemia or established renal failure [11]. In the current case, 3000 mL/day of intravenous fluids, febuxostat (20 mg/day orally), and intravenous furosemide (20 mg/day) were effective in stabilizing the patient’s condition, and renal replacement therapy was not required.

Another clinically relevant issue is whether rechallenging the causative agent is feasible after TLS resolution. While successful rechallenges with immune checkpoint inhibitors have been reported following immune-related adverse events [12], no published data exist regarding rechallenge of molecular targeted therapies after TLS. In this case, selpercatinib was resumed with close biochemical monitoring and without recurrence of TLS, suggesting that, in selected patients, retreatment may be possible with caution.

The expanding use of targeted therapies in oncology has reshaped treatment paradigms and improved outcomes across numerous tumor types. However, the potential for these agents to cause rare but severe toxicities, such as TLS, must not be overlooked. Importantly, TLS may occur even in patients who do not exhibit elevated LDH, renal dysfunction, or bulky disease. The present case underscores the need for early laboratory surveillance and risk awareness when initiating treatment with agents that exhibit rapid tumoricidal activity.

In summary, TLS can occur in patients with RET fusion-positive NSCLC treated with selpercatinib, even in the absence of conventional TLS risk factors. Early biochemical monitoring and prophylactic measures should be considered when initiating highly effective molecular targeted therapies. Furthermore, this case provides preliminary evidence that rechallenge may be feasible after TLS resolution, although further data are needed to guide clinical decision-making in such scenarios.

## Conclusions

TLS can occur in RET fusion-positive NSCLC patients treated with selpercatinib, even without conventional risk factors. Early monitoring and prophylactic measures should be considered when initiating highly active targeted therapies. This case also suggests that, with appropriate management, rechallenge may be possible, though further studies are needed to guide such decisions.
